# Unraveling How Antimicrobial Lipid Mixtures Disrupt Virus-Mimicking Lipid Vesicles: A QCM-D Study

**DOI:** 10.3390/biomimetics9020067

**Published:** 2024-01-24

**Authors:** Suji Moon, Tun Naw Sut, Bo Kyeong Yoon, Joshua A. Jackman

**Affiliations:** 1 School of Chemical Engineering and Translational Nanobioscience Research Center, Sungkyunkwan University, Suwon 16419, Republic of Korea; 2 School of Healthcare and Biomedical Engineering, Chonnam National University, Yeosu 59626, Republic of Korea

**Keywords:** antimicrobial lipid, fatty acid, monoglyceride, vesicle, critical micelle concentration, quartz crystal microbalance–dissipation

## Abstract

Single-chain lipid amphiphiles such as fatty acids and monoglycerides are promising antimicrobial alternatives to replace industrial surfactants for membrane-enveloped pathogen inhibition. Biomimetic lipid membrane platforms in combination with label-free biosensing techniques offer a promising route to compare the membrane-disruptive properties of different fatty acids and monoglycerides individually and within mixtures. Until recently, most related studies have utilized planar model membrane platforms, and there is an outstanding need to investigate how antimicrobial lipid mixtures disrupt curved model membrane platforms such as intact vesicle adlayers that are within the size range of membrane-enveloped virus particles. This need is especially evident because certain surfactants that completely disrupt planar/low-curvature membranes are appreciably less active against high-curvature membranes. Herein, we conducted quartz crystal microbalance–dissipation (QCM-D) measurements to investigate the membrane-disruptive properties of glycerol monolaurate (GML) monoglyceride and lauric acid (LA) fatty acid mixtures to rupture high-curvature, ~75 nm diameter lipid vesicle adlayers. We identified that the vesicle rupture activity of GML/LA mixtures mainly occurred above the respective critical micelle concentration (CMC) of each mixture, and that 25/75 mol% GML/LA micelles exhibited the greatest degree of vesicle rupture activity with ~100% efficiency that exceeded the rupture activity of other tested mixtures, individual compounds, and past reported values with industrial surfactants. Importantly, 25/75 GML/LA micelles outperformed 50/50 GML/LA micelles, which were previously reported to have the greatest membrane-disruptive activity towards planar model membranes. We discuss the mechanistic principles behind how antimicrobial lipid engineering can influence membrane-disruptive activity in terms of optimizing the balance between competitive membrane remodeling processes and inducing anisotropic vs. isotropic spontaneous curvature in lipid membrane systems.

## 1. Introduction

Single-chain lipid amphiphiles include important biomolecules such as fatty acids and monoglycerides and are receiving attention as promising antimicrobial agents to disrupt membrane-enveloped pathogens such as bacteria and viruses [1,2]. While conventional antibacterial and antiviral agents typically inhibit specific proteins that are critical to key steps in pathogen life cycles [3], fatty acids and monoglycerides—also known as antimicrobial lipids—exhibit broad-spectrum inhibitory activity by causing phospholipid membrane disruption [4,5]. In the antibacterial context, bacterial cell membrane inhibition can result in cell death or loss of cellular functions depending on the extent of membrane disruption, which is related to membrane permeability changes and/or membrane lysis [6,7]. On the other hand, in the antiviral context, viral membrane disruption compromises the structural integrity of membrane-enveloped virus particles extracellularly, in turn preventing cell infection and reducing viral load [8]. With this broad scope of inhibitory activities, antimicrobial lipids have been widely utilized in various industrial applications across the healthcare, cosmetics, skincare, food preservation, and agriculture sectors [9,10].

To guide compound selection, structure–function relationship studies have investigated how antimicrobial lipid properties such as chain length and headgroup charge affect antimicrobial potency, which has led to identifying that twelve-carbon-long lauric acid (LA) and glycerol monolaurate (GML) are among the most active saturated fatty acids and monoglycerides, respectively [11,12,13]. However, since biological assays are often based on endpoint-related outputs such as measuring loss of cell viability or infectivity [14], they offer limited mechanistic insights into the corresponding membrane-disruptive processes from a biomacromolecular interaction perspective. Biophysical measurement strategies based on membrane-mimicking phospholipid bilayer platforms are compatible with different biosensing techniques to track real-time interactions and offer a complementary approach to gain mechanistic understanding [15]. By studying the interaction between antimicrobial lipids and biomimetic membrane platforms [e.g., planar supported lipid bilayers (SLBs)], mechanistic insights into how antimicrobial lipids disrupt membranes can be tracked in real time and correlated with information such as the critical micelle concentration (CMC) of an antimicrobial lipid [16,17]. Such approaches have provided a molecular-level explanation to rationalize why certain antimicrobial lipids are more potent than other ones (e.g., due to a lower CMC) while also revealing how certain mixtures of antimicrobial lipids can exhibit synergistic membrane disruption [18]. In particular, an equimolar (1:1) mixture of GML and LA has been reported to demonstrate more extensive membrane disruption of a planar SLB compared to GML or LA alone, as indicated by around three-fold greater membrane lysis [18]. Of note, the specific GML/LA molar ratio was found to be a more impactful determinant than the total GML/LA concentration, thus highlighting the importance of precisely tuning the antimicrobial lipid composition. 

In general, many related biophysical studies have been conducted on planar model membrane platforms such as SLBs [15,19] and tethered lipid bilayer membranes [20], while extending such studies to more complex model membrane platforms is important because recent evidence has shown that membrane nanoarchitecture features such as curvature can play critical roles in modulating biomacromolecular interaction processes [21]. For example, sodium dodecyl sulfate (SDS) surfactant micelles are known to cause the rapid, complete membrane solubilization of planar SLBs, but they have been shown to be appreciably less active against model membrane platforms consisting of highly curved, sub-100 nm lipid vesicles (only ~60% solubilization against small, ~70 nm diameter vesicles vs. ~100% solubilization against larger, ~120 nm diameter vesicles and planar SLBs) that resemble the size of enveloped virus particles [22]. This finding supports that the micellar aggregation of antimicrobial lipids and surfactants is not the only predictor of antimicrobial potency and reinforces the potential of rationally developing antimicrobial lipid mixtures for different application scopes [23]. Given the high antimicrobial activity of GML and LA individually and the previously reported synergistic membrane disruption exhibited by GML/LA mixtures against planar SLBs [18], understanding how GML/LA mixtures disrupt highly curved, sub-100 nm lipid vesicles would be advantageous, especially if they could be optimized to outperform traditionally used surfactants such as SDS.

Herein, we investigated the real-time interactions between GML/LA mixtures and intact vesicle platforms consisting of a close-packed adlayer of ~75 nm diameter, zwitterionic 1,2-dioleolyl-*sn*-glycero-3-phosphocholine (DOPC) lipid vesicles. The vesicle size range was selected to be around the range of medically important enveloped viruses and also corresponds to the vesicle size range in which SDS was reported to be less effective [22]. While DOPC lipid vesicles are simplified mimics of more compositionally complex viral envelopes, they provide a well-controlled model system to study curvature-related membrane interactions and have been previously used to characterize the membrane-disruptive properties of antiviral peptides with similar rupture efficiencies compared to vesicles with more complex lipid compositions [24]. The quartz crystal microbalance–dissipation (QCM-D) technique was utilized as the main measurement tool to track corresponding changes in the acoustic mass and viscoelastic properties of the intact vesicle platform when GML/LA mixtures were added. The frequency (Δf) and energy dissipation (ΔD) signals of the oscillating QCM-D sensor chip upon which the intact vesicle platform was fabricated were temporally tracked in a label-free format and provide information related to the mass and viscoelastic properties of the adlayer, respectively [25,26]. Particular focus was placed on evaluating the extent to which different mixtures induced membrane morphological changes during the interaction process along with the resulting vesicle disruption efficiency. This approach led us to identify specific GML/LA mixtures that efficiently disrupted sub-100 nm lipid vesicles to a greater extent than GML or LA alone. Interestingly, the optimal GML/LA ratio to effectively disrupt vesicles in this case was different from the previously identified optimal ratio to disrupt planar SLBs [18], which further underscores the importance of taking into account a membrane nanoarchitecture perspective [27,28] for antimicrobial lipid engineering. 

## 2. Materials and Methods

### 2.1. Reagents 

LA, 1-pyrenecarboxaldehyde, and general chemical reagents were obtained from Sigma-Aldrich (St. Louis, MO, USA), while GML was procured from Abcam (Cambridge, UK). DOPC lipids in chloroform were supplied by Avanti Polar Lipids (Alabaster, AL, USA). Tris buffer solution was prepared by dissolving 10 mM Tris and 150 mM NaCl in deionized water (>18 MΩ·cm) (MilliporeSigma, Burlington, MA, USA), and the pH was adjusted to 7.5. 

### 2.2. Vesicle Preparation

DOPC lipid vesicles were prepared using the extrusion method, as previously described [29]. First, DOPC lipids were dried with nitrogen gas in a glass vial to form a lipid film on the sidewall. Then, the glass vial was placed overnight in a desiccator to evaporate residual chloroform. Next, the dry lipid film was hydrated in Tris buffer to a 5 mg/mL lipid concentration (~6.4 mM) and then vortexed for 3 min. The vesicles were extruded by passing the hydrated lipid suspension through polycarbonate membranes with 50 nm diameter pores for a total of 31 times by using a mini-extruder apparatus (Avanti Polar Lipids). The size distribution of the extruded lipid vesicles was determined by dynamic light scattering (DLS) measurements with the ELSZ-2000 instrument (Otsuka Electronic Co., Ltd., Osaka, Japan), and the mean vesicle diameter was ~75 nm and the polydispersity index was ~0.1. Immediately before the experiment, the vesicles were diluted to 0.1 mg/mL (~127 µM). 

### 2.3. GML/LA Mixture Preparation

Stocks of 200 mM GML and LA were individually dissolved in pure ethanol. Each ethanol stock solution was then diluted in buffer to the respective molar ratio concentration required for equi-volume mixing to achieve a 2 mM total GML/LA concentration. Prior to mixing, the aqueous GML and LA solutions were heated in a 70 °C water bath for 30 min, followed by mixing and then extensive vortexing. Further buffer dilution steps were taken to reach the final desired mixture concentration, and all samples were freshly prepared before experiments.

### 2.4. Critical Micelle Concentration (CMC) Assay

The critical micelle concentration (CMC) values of GML/LA mixtures were determined by taking wavelength-shift spectroscopy measurements with a SpectraMax iD5 microplate reader (Molecular Devices, San Jose, CA, USA), as previously described [30,31]. First, 50 μM 1-pyrenecarboxaldehyde was prepared in methanol and added to a glass vial, which was then left in a fume hood to evaporate the methanol. Next, the dried fluorescent probe was hydrated to a final 0.1 μM concentration in buffer solution containing the desired total GML/LA concentration, followed by vortexing. Thus, the probe concentration was fixed at 0.1 μM, while the total GML/LA concentration was varied for each GML/LA molar ratio (100/0, 75/25, 50/50, 25/75, 0/100). Experimentally, the excitation wavelength was set at 366 nm, and the fluorescence emission spectrum was scanned from 410 nm to 600 nm. The measurements were conducted at room temperature, and at least four technical replicates were performed for each data point.

### 2.5. Quartz Crystal Microbalance–Dissipation (QCM-D)

QCM-D measurements were performed using a Q-Sense E4 instrument (Biolin Scientific AB, Gothenburg, Sweden), as previously described [25]. Prior to each round of experiments, TiO_2_-coated sensor chips (model no. QSX 310, Biolin Scientific AB) were cleaned with deionized water and ethanol and then dried with nitrogen, followed by 1 min oxygen plasma treatment in a CUTE-1MPR machine (Femto Science Inc., Hwaseong, Republic of Korea). During the experiments, the sample solutions were added into the chambers using a peristaltic pump (Reglo Digital, Ismatec, Glattbrugg, Switzereland) at a defined flow rate of 100 μL/min. The QSoft (version no. 2.5.28.732) and QTools (version no. 3.1.33) software programs (Biolin Scientific AB, Gothenburg, Sweden) were used to complete data collection at multiple overtones and data processing, respectively. All presented QCM-D data were collected from the 5th overtone, and at least three independent replicates were performed per condition. For statistical analysis, two-tailed Student’s *t*-tests were performed using the GraphPad Prism software package (version no. 10.1.2; Boston, MA, USA), and *p* < 0.05, *p* < 0.01, and *p* < 0.001 indicate the levels of statistical significance (*, **, ***).

## 3. Results and Discussion

### 3.1. Study Design

**Figure 1:**
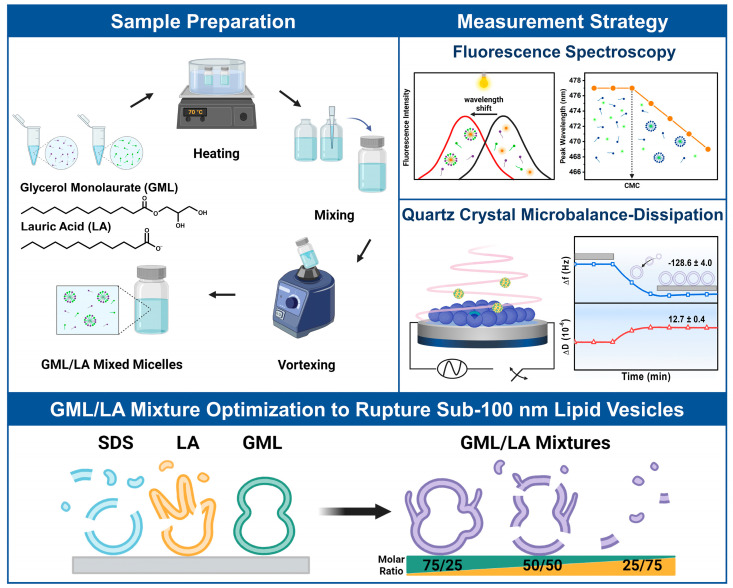
Overview of measurement strategy and experimental objective. GML and LA buffer solutions were prepared individually, followed by mixing and vortexing to prepare GML/LA mixtures of defined molar ratio. GML/LA mixtures had different CMC values depending on the molar ratio, as determined by concentration-dependent fluorescence spectroscopy experiments that detected fluorescent probe partitioning into micelle interiors. QCM-D experiments were performed to characterize the interactions of GML/LA mixtures, above and below CMC, with DOPC lipid vesicle adlayers in order to characterize membrane-disruptive interactions and to identify antimicrobial lipid mixtures that exhibit more efficient vesicle disruption activity than individual antimicrobial lipids or related surfactants.

We designed our experiments to investigate the interactions of GML/LA mixtures with intact DOPC lipid vesicle adlayers depending on the GML/LA molar ratio. The experimental scope included solution-phase CMC measurements of the tested GML/LA ratios with wavelength-shift fluorescence spectroscopy to define test concentrations above and below CMC, followed by QCM-D experiments to investigate how the different GML/LA mixtures interact with intact vesicle adlayers on TiO_2_-coated sensor surfaces (Figure 1). Five representative mixtures were selected in 25 mol% molar ratio increments to form GML/LA mixtures ranging from 100/0 to 0/100 mol% GML/LA. 

For the CMC measurements, a hydrophobic probe molecule (1-pyrenecarboxaldehyde) with fluorescence emission properties was mixed with different GML/LA concentrations (at defined molar ratios), whereby the probe partitioned into the hydrophobic interior of micelles when the CMC was reached. Probe partitioning was detected by a drop in the peak emission wavelength due to different dielectric environments in the hydrophobic micelle interior vs. in aqueous solution. The total GML/LA concentration immediately prior to the drop point was defined as the CMC for that particular GML/LA ratio, which allowed us to define test concentrations above and below the CMC for the QCM-D experiments.

For the QCM-D measurements, intact vesicle adlayers were first assembled on TiO_2_-coated sensor surfaces in situ, and corresponding changes in the resonance frequency (Δf) and energy dissipation (ΔD) signals relative to buffer baseline values were tracked in real time. The Δf and ΔD signals are related to the hydrodynamically coupled mass and viscoelastic properties of the adsorbate, respectively [25]. At the fabrication stage, the QCM-D responses corresponded to the adsorption of the ~75 nm diameter DOPC lipid vesicles, which were used in this study, relative to the buffer baseline. The resulting Δf and ΔD shifts were around −128.6 ± 4.0 Hz and 12.7 ± 0.4 × 10^−6^, respectively, which agree well with the literature values for intact vesicle adlayers on TiO_2_ surfaces [32] and support that the vesicles adsorbed and remained intact without rupture as expected. Afterwards, the QCM-D measurements were continued, and different GML/LA mixtures (defined ratio and concentration) were added to the intact vesicle adlayer platform, and resulting interactions were tracked. Particular focus was placed on the interaction kinetics and extent of membrane disruption due to the addition of GML/LA mixtures (labeled as treatment) and after a subsequent buffer washing step (labeled as post-washing). Note that in the QCM-D data presented below, the elapsed measurement time was reset to zero after vesicle adsorption so that the initial Δf and ΔD shift values at *t* = 0 min correspond to the vesicle adlayer platform, and the appropriate GML/LA mixture was added from *t* = 5 min onwards.

### 3.2. CMC Characterization of GML/LA Mixtures and QCM-D Verification

We began by determining the CMC values of the different GML/LA mixtures, which allowed us to define experimental concentrations for subsequent QCM-D experiments because it is known that antimicrobial lipids mainly disrupt phospholipid bilayers in the micellar state at and above CMC, whereas they are typically less active or inactive as monomers below CMC [33,34]. 

**Figure 2:**
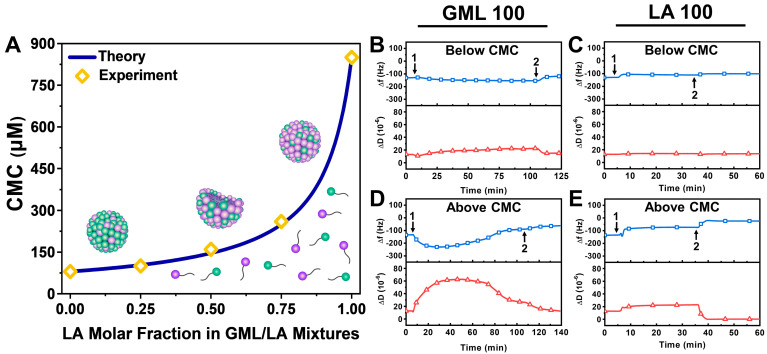
Characterization of CMC values for GML/LA mixed micelles and QCM-D verification of GML and LA vesicle disruption effects. (**A**) CMC values of GML/LA mixtures as a function of LA molar fraction determined based on experimentally determined values (yellow diamonds) and theoretical calculations (blue line). (**B**–**E**) QCM-D resonance frequency (Δf) and energy dissipation (ΔD) shifts as a function of time for GML and LA interactions with an intact vesicle adlayer composed of ~75 nm diameter, DOPC lipid vesicles. Time-lapse QCM-D data corresponding to below CMC (0.5× CMC) were recorded for (**B**) GML and (**C**) LA. Similar data above CMC (2× CMC) were also recorded for (**D**) GML and (**E**) LA. The baseline values correspond to the intact vesicle adlayer on the TiO_2_-coated sensor surface, and GML or LA were injected starting at *t* = 5 min (arrow 1), followed by buffer washing step (arrow 2). QCM-D data are representative of at least three independent measurements per condition.

For each tested GML/LA ratio, the fluorescence emission spectrum of the 1-pyrenecarboxaldehyde probe in different total GML/LA concentrations was measured in order to determine the CMC at that particular ratio [35]. Figure 2A presents the measured CMC values as a function of the GML/LA ratio. As expected, the CMC values of 100/0 GML/LA and 0/100 GML/LA were determined to be around 80 μM and 850 μM, respectively, whereas 75/25, 50/50, and 25/75 GML/LA had CMC values of 100 μM, 160 μM, and 260 μM, respectively. This observed trend is consistent with a greater LA fraction causing more extensive intermolecular repulsion within self-assembled micelles and hence yielding a higher CMC, whereas the nonionic headgroup of GML makes it more favorable for GML/LA mixed micelles to form at a lower CMC when the GML fraction is relatively higher [36].

For comparison, we also plotted the theoretically predicted CMC of the GML/LA mixed micelles at different LA fractions by assuming the ideal mixing of GML and LA molecules (no net interaction) according to a pseudo-phase separation model [37]. According to this model, the CMC value of a binary GML/LA mixture can be expressed as $C_{\mathrm{Mix}}^{*}$ and is defined by the respective CMC values of GML and LA, namely $C_{\mathrm{GML}}$ and $C_{\mathrm{LA}}$, as follows [38]:

$$\frac{1}{C_{\mathrm{Mix}}^{*}}=\frac{\left(1-\alpha \right)}{C_{\mathrm{GML}}}+\frac{\alpha }{C_{\mathrm{LA}}}$$

where α is the molar fraction of one component (defined to be LA in this case) and 1 − α is the molar fraction of the other component (GML in this case). 

Next, we conducted QCM-D experiments to verify the membrane-disruptive interactions of GML and LA as controls before proceeding to test the GML/LA mixtures. Note that the QCM-D measurement signals at *t* = 0 min correspond to already fabricated, intact vesicle adlayers with Δf and ΔD shifts around −128.6 ± 4 Hz and 12.7 ± 0.4 × 10^−6^, respectively, relative to the initial buffer baselines (cf. Figure 1). All subsequent Δf and ΔD shifts are also reported relative to the initial buffer baseline (i.e., before vesicle adsorption). Below the CMC, both GML and LA had nearly negligible interactions with intact vesicle adlayers. The addition of 40 μM GML resulted in a slight decrease in the Δf signal to around −154 Hz and a corresponding increase in the ΔD signal to around 23 × 10^−6^, which indicates compound binding and some degree of vesicle swelling (Figure 2B). Upon subsequent buffer washing, the Δf signal went back up to around −119 Hz, and the ΔD signal returned to around 15 × 10^−6^. On the other hand, the addition of 425 μM LA caused a slight increase in the Δf signal to around −111 Hz, which further increased to around −101 Hz upon buffer washing; however, there were nearly negligible changes in the ΔD signal in that case (Figure 2C). 

By contrast, the addition of GML and LA above the CMC caused extensive disruption of intact vesicle adlayers. The addition of 160 μM GML caused a gradual but marked decrease in the Δf signal to around −230 Hz, which was accompanied by a large increase in the ΔD signal to around 63 × 10^−6^ (Figure 2D). Subsequent buffer washing led to final Δf and ΔD shifts around −64 Hz and 13 × 10^−6^, respectively, which supports that GML initially caused membrane budding-like morphological changes followed by partial disruption upon buffer washing [22]. By contrast, upon 1700 μM LA addition, the Δf signal increased to around −74 Hz and the ΔD signal increased to 23 × 10^−6^, while subsequent buffer washing caused a further increase in the Δf signal to around −24 Hz and a decrease in the ΔD signal to around ~0.3 × 10^−6^ (Figure 2E). The QCM-D shift magnitudes and corresponding kinetics are consistent with the membrane-disruptive effects of LA on intact vesicle adlayers and point to extensive solubilization [22]. These QCM-D results verify the CMC-dependent effects of GML and LA on intact vesicle adlayers in line with the recorded CMC values from the fluorescence spectroscopy experiments and allowed us to proceed with GML/LA mixture testing.

### 3.3. Interactions of GML/LA Mixtures with Intact Vesicle Adlayers

**Figure 3:**
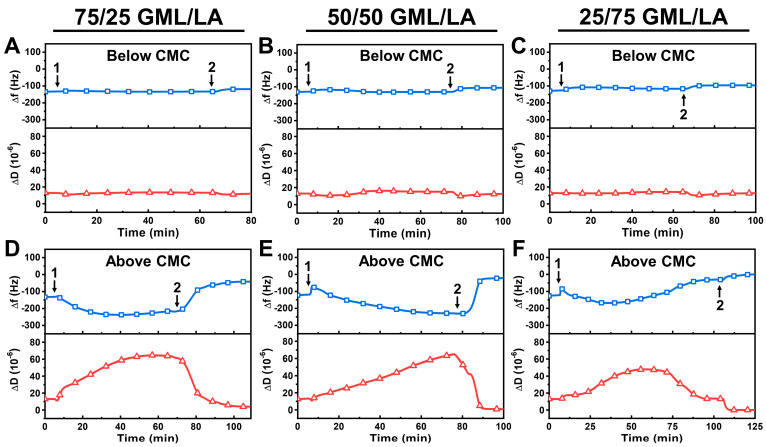
QCM-D characterization of GML/LA mixture interactions with intact vesicle adlayer. QCM-D resonance frequency (Δf) and energy dissipation (ΔD) shifts as a function of time for GML/LA mixture interactions with an intact DOPC lipid vesicle adlayer. Time-lapse QCM-D data corresponding to below CMC (0.5× CMC) were recorded for (**A**) 75/25 GML/LA, (**B**) 50/50 GML/LA, and (**C**) 25/75 GML/LA mixtures. (**D**–**F**) Equivalent QCM-D data were obtained for GML/LA mixtures above CMC (2× CMC) as well. The baseline values correspond to the intact vesicle adlayer on the TiO_2_-coated sensor surface, and the GML/LA mixtures were injected starting at *t* = 5 min (arrow 1), followed by buffer washing step (arrow 2). Graphs are representative of at least three independent measurements per condition.

We continued the QCM-D measurements with GML/LA mixtures at concentrations below and above the respective CMC values (Figure 3). Accordingly, we tested the following concentrations: 50 μM and 200 μM for 75/25 GML/LA, 80 μM and 320 μM for 50/50 GML/LA, and 130 μM and 520 μM for 25/75 GML/LA. Below CMC, all the tested GML/LA monomers were largely inactive against the intact vesicle adlayers, as indicated by nearly negligible QCM-D signal shifts during the interaction and after buffer washing (Figure 3A–C).

On the other hand, the GML/LA mixtures above the CMC exhibited appreciable membrane-disruptive activities. In the case of 75/25 GML/LA micelles, the Δf signal decreased to around −237 Hz and the ΔD signal increased to around 65 × 10^−6^, and both signals stabilized around these respective values (Figure 3D). Upon buffer washing, the Δf signal increased to around −42 Hz and the ΔD signal decreased to around 4 × 10^−6^, which indicate extensive but incomplete vesicle disruption. Similarly, the addition of 50/50 GML/LA micelles caused the Δf signal to exhibit a small, transient spike and then decrease gradually to around −230 Hz, where it nearly stabilized, while the ΔD signal increased up to around 64 × 10^−6^ (Figure 3E). However, upon buffer washing, the Δf signal increased rapidly to around −22 Hz and the ΔD signal also decreased to around 1 × 10^−6^, which indicate more extensive vesicle disruption compared to the 75/25 GML/LA micelle case, but the disruption effect was still incomplete. Since the final lipid adlayer had a low ΔD signal (~1 × 10^−6^ or lower), the Sauerbrey model [39] could be applied to convert the Δf signal into the surface mass density (Δm), which was estimated at ~389 ng/cm^2^. This value is appreciably lower than the surface mass density for an adlayer of intact vesicles with a similar size, which is typically around at least 4000 ng/cm^2^ or higher [40], and this difference points to extensive disruption of the intact vesicle adlayer.

In marked contrast to the preceding two cases, the addition of 25/75 GML/LA micelles caused extensive membrane disruption during the GML/LA mixture addition step itself, even prior to the buffer washing step. Initially, upon 25/75 GML/LA micelle addition, there was a small, transient spike followed by a downward shift in the Δf signal to around −169 Hz that was accompanied by an increase in the ΔD signal to around 48 × 10^−6^, before the Δf and ΔD signals reached inflection points and stabilized at around −30 Hz and 13 × 10^−6^, respectively (Figure 3F). After the buffer washing step, the Δf and ΔD signals reached final values around ~0 Hz and ~0 × 10^−6^, respectively, which indicated complete removal of the vesicle adlayer from the sensor surface (i.e., ~0 ng/cm^2^ of adsorbed lipid molecules according to the Sauerbrey model). Together, these results support that 25/75 GML/LA micelles caused complete membrane solubilization of the vesicle adlayer, while 50/50 and 75/25 GML/LA micelles only caused partial solubilization.

**Figure 4:**
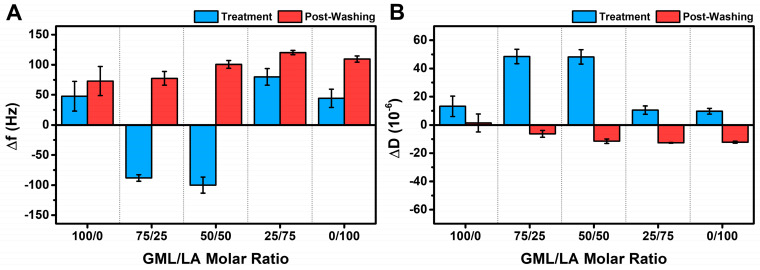
Maximum QCM-D shift responses during GML/LA micelle interaction step and post-washing. Changes in (**A**) Δf and (**B**) ΔD shifts are reported during micelle addition step (labeled as Treatment, blue) and after buffer washing step (labeled as Post-Washing, red). All data were obtained at 2× CMC and are reported as net values relative to the QCM-D shift values for an intact vesicle adlayer (prior to micelle addition). The results are reported as the mean ± standard deviation from at least *n* = 3 independent measurements.

To rationalize this trend in terms of the vesicle interaction behavior, we first discuss the maximum QCM-D responses that occurred during the GML/LA micelle addition step and after the final buffer washing step for all the tested GML/LA mixtures as well as for the LA and GML controls (all at 2× CMC). Figure 4A presents the net changes in the Δf signal during the interaction (i.e., treatment step due to micelle addition; blue color) and after the final buffer washing step (i.e., post-washing; red color). The corresponding net changes in the ΔD signal are presented in Figure 4B, and all reported changes in both graphs are relative to the intact vesicle adlayer’s QCM-D values (cf. Δf ~−128.6 Hz and ΔD ~12.7 × 10^−6^ on average) prior to GML/LA micelle addition.

In terms of the net Δf shifts, GML caused +48 ± 25 Hz and +73 ± 24 Hz changes due to micelle addition and after buffer washing, respectively, relative to the specific intact vesicle adlayer shift value for each experiment. These positive Δf shifts indicate that vesicle disruption was the main interaction effect, whereas marked differences occurred in the cases of 75/25 and 50/50 GML/LA micelle treatment. In those latter two cases, micelle addition caused net Δf shifts around −90 to −100 Hz, which indicated that the major interaction effect was membrane budding-like behavior, i.e., mainly due to an increase in hydrodynamically coupled solvent. Subsequent buffer washing caused large changes in the Δf signal, and the final net Δf shifts were around +77 to +100 Hz above the intact vesicle adlayer shift value, which indicated resulting vesicle disruption only after buffer washing.

Interestingly, in the case of 25/75 GML/LA micelle treatment, the initial micelle addition step caused a net Δf shift of around +80 ± 14 Hz—an indication of direct vesicle interaction—while subsequent buffer washing caused a final net Δf shift of around +120 ± 4 Hz. This finding underscores the importance of tuning the particular GML/LA molar ratio because adjusting the mixed micelle composition had dramatic effects on the vesicle interaction behavior, shifting the interaction effect from membrane budding with 75/25 and 50/50 GML/LA micelles to direct vesicle disruption with 25/75 GML/LA micelles. Similarly to GML, LA addition caused a net Δf shift of around +44 ± 15 Hz, and subsequent buffer washing caused a final net Δf shift of around +110 ± 5 Hz. In addition to the Δf shift trends, the net ΔD shifts due to the addition of LA, GML, and 25/75 GML/LA micelles were less than 20 × 10^−6^, whereas the addition of 75/25 and 50/50 GML/LA micelles caused net ΔD shifts greater than 40 × 10^−6^. These differences are consistent with membrane budding effects (i.e., relatively larger ΔD shifts) vs. direct vesicle disruption (i.e., relatively smaller ΔD shifts). After buffer washing, the final net ΔD shifts tended to progressively decrease with increasing LA fraction, with the largest shift decrease observed for the 25/75 GML/LA micelle case and the second largest shift decrease for the LA micelle case.

### 3.4. Vesicle Rupture Efficiency Evaluation

**Figure 5:**
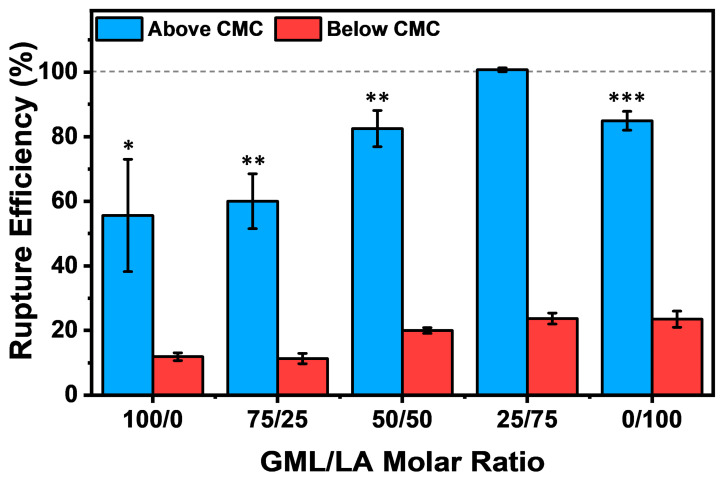
Quantitative evaluation of vesicle rupture efficiency by GML/LA mixtures. The extent of vesicle disruption was expressed in terms of lipid removal from the sensor surface and computed from the final Δf shift after GML/LA mixture addition and buffer washing vs. the Δf shift of the intact vesicle adlayer prior to GML/LA mixture addition. All Δf shifts were recorded relative to the buffer baseline signal prior to vesicle adsorption on the TiO_2_-coated sensor surface. The data are reported as a function of the GML/LA molar ratio, and the mean and standard deviation were computed from at least *n* = 3 independent measurements. For the above CMC data (2× CMC), markers denote the statistical significance of individual groups compared to the 25/75 GML/LA group. The asterisks, *, **, and *** correspond to *p* < 0.05, *p* < 0.01, and *p* < 0.001, respectively.

To gain quantitative insights into the relative extent of vesicle disruption, we also evaluated the vesicle rupture efficiency of the different GML/LA mixtures above and below CMC based on the measured changes in the QCM-D Δf signal (Figure 5). The rupture efficiency was determined based on lipid adlayer removal from the TiO_2_-coated sensor surface, whereby 100% rupture efficiency corresponds to the complete removal of adsorbed lipid molecules from the sensor surface and 0% rupture efficiency corresponds to a negligible effect on lipid removal from the sensor surface. The rupture efficiency was quantified as follows:

$$\mathrm{Rupture}\;\mathrm{Efficiency}\;\%=\left(\frac{\Delta f_{\mathrm{vesicle}}-\Delta f_{\mathrm{final}}}{\Delta f_{\mathrm{vesicle}}}\right)\times 100\%$$

where Δf_vesicle_ is the Δf shift corresponding to the intact vesicle adlayer prior to GML/LA mixture addition, and Δf_final_ is the Δf shift corresponding to the final value after GML/LA mixture addition and buffer washing. Both Δf shift values were calculated relative to the initial buffer baseline signal prior to vesicle addition, and negative values due to compound binding were considered to be ~0% since no lipid removal was detected in those cases.

Based on this approach, at 2× CMC, the 100/0 and 75/25 GML/LA mixtures exhibited a moderate degree of vesicle disruption, as evidenced by rupture efficiency values around 55.6 ± 17.4% and 60.0 ± 8.5%, respectively. By contrast, the 50/50 GML/LA mixture demonstrated enhanced vesicle disruption, and the rupture efficiency value in that case was 82.5 ± 5.6%, which is consistent with past findings that the equimolar GML/LA mixture had high membrane-disruptive activity against planar SLBs compared to other mixtures and GML or LA alone [18]. Interestingly, in the present intact vesicle adlayer case, we also observed that the 25/75 GML/LA mixture induced an even greater level of vesicle disruption, which translated into a rupture efficiency of 100.7 ± 0.6% and indicated complete membrane solubilization. By contrast, the 0/100 GML/LA mixture had a rupture efficiency of 84.9 ± 2.9%, which is similar to the equimolar mixture treatment effect. Collectively, these findings support that the 25/75 GML/LA mixture had the greatest membrane-disruptive effect against the intact vesicle adlayer. On the other hand, at 0.5× CMC, all the GML/LA mixtures caused minimal vesicle disruption, as demonstrated by rupture efficiency values of less than 25% and verifying the importance of micellar self-assembly. 

From these data, we can conclude that all the tested GML/LA mixtures were mainly active in the micellar state, and that the 25/75 GML/LA mixture caused the greatest degree of vesicle rupture. The aforementioned rupture efficiency values were calculated after a final buffer washing step to remove weakly adsorbed lipid species, while we also performed similar rupture efficiency calculations to further evaluate the degree of vesicle disruption after GML/LA micelle addition (at 2× CMC) and prior to the final buffer washing step. In that case, the rupture efficiency values for GML and LA alone were 36.6 ± 18.1% and 34.3 ± 11.1%, respectively, whereas the corresponding values for 75/25, 50/50, and 25/75 GML/LA mixtures were ~0%, ~0%, and 67.1 ± 10.2%, respectively. As discussed before in the SLB context [18], these findings support that the GML/LA molar ratio is the major determinant of membrane disruption rather than the total GML/LA concentration, i.e., greater disruption could be observed in some cases where the total GML/LA concentration was lower depending on the molar ratio.

At the same time, there are important differences between the past SLB data and current intact vesicle data that warrant attention. Notably, the optimal GML/LA ratio that caused the greatest membrane disruption of vesicles was different than the optimal ratio for SLB disruption determined in a past study [18]. Previously, it was reported that the 50/50 GML/LA mixture caused the greatest level of membrane disruption against SLBs, whereas GML or LA alone had the lowest levels. In marked contrast, we observed that the 25/75 GML/LA mixture caused the greatest level of membrane disruption against intact vesicles. This difference is noteworthy because our results further indicate that, while GML alone exhibited the lowest level of vesicle disruption that was comparable to its effects on SLBs, LA alone caused a relatively greater level of vesicle disruption compared to its effects on SLBs post-washing. This finding suggests that LA causes greater membrane disruption of curved membranes compared to planar membranes, whereas GML had similar effects on both model membrane types.

To rationalize the greater disruptive effects of LA on curved membranes and implications for GML/LA mixture optimization, it should be remarked that the lipid bilayer leaflets in highly curved membranes such as sub-100 nm vesicles are already strained (prior to GML/LA addition) due to the geometrical packing of the phospholipid molecules [41] (i.e., greater area per lipid in the outer leaflet and smaller area per lipid in the inner leaflet compared to planar lipid bilayers). Also, while experimentally determined membrane partition coefficient values of GML and LA have not been reported to our knowledge, the two compounds have similar octanol–water partition coefficients (*P*) in the range of ~10^4^, which have been discussed in relation to membrane-disruptive properties [42,43]. Molecular dynamics (MD) simulations have also estimated the Gibbs free energy (Δ*G*) values associated with DOPC lipid membrane partitioning, which were determined to be around −34 and −22 kJ/mol for GML and LA, respectively [44]. These computational estimates suggest that GML membrane partitioning is more thermodynamically favorable than that of LA, while the type of membrane disruption also depends on the membrane translocation properties of the partitioned compounds across the bilayer leaflets. Under the test conditions, GML is nonionic and LA is anionic, which makes it more thermodynamically favorable for GML to translocate than LA [45]. MD simulations have further estimated the Δ*G* values associated with DOPC lipid membrane translocation, which were determined to be around +23 and +44 kJ/mol for GML and LA, respectively [44].

Accordingly, since LA has a negatively charged headgroup and thus a lower rate of membrane translocation between the two leaflets due to the higher energy barrier [44,45], the intercalation of LA molecules into the vesicle bilayer induces anisotropic spontaneous curvature in the vesicle bilayer, and its insertion mainly causes greater strain in the outer leaflet [46]. This effect is evident from the rapid, albeit still incomplete, disruption kinetics observed in the QCM-D measurements for the LA case (cf. Figure 2). By contrast, GML has a higher rate of membrane translocation across the two leaflets and thus mainly induces isotropic spontaneous curvature, which results in more gradual vesicle disruption, as observed in the measured interaction kinetics. Together, these findings support that the combination of the geometry-related strain of lipids in the outer leaflet plus the strain enhancement caused by LA insertion contributes to heightened vesicle disruption in the LA case, especially in combination with shear flow during the buffer washing step. It has also been noted that the DOPC-LA lateral interaction is modestly larger than the DOPC-GML lateral interaction [18], which may further contribute to this effect. Even so, and despite a lower total concentration, the 25/75 GML/LA mixture still exhibited greater vesicle disruption than LA alone, which can be attributed to the competition that arises between GML- and LA-induced membrane morphological changes in the vesicle structure and causes synergistic membrane disruption [21].

**Figure 6:**
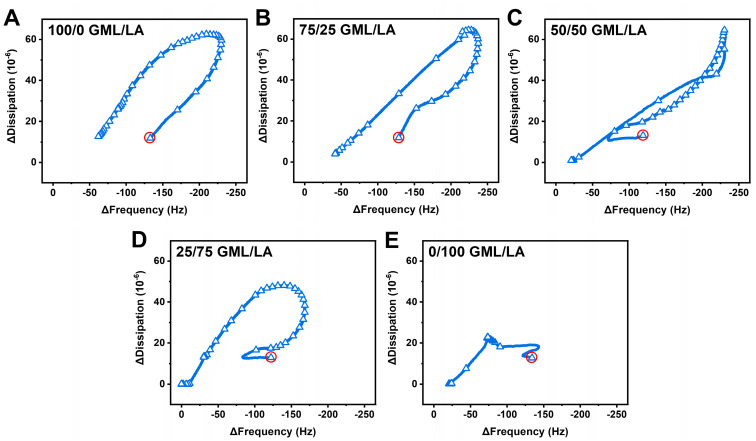
Time-independent plots of QCM-D shift responses during GML/LA micelle interaction step and post-washing. Measured ΔD shifts are plotted as a function of Δf shifts for (**A**) 100/0 GML/LA, (**B**) 75/25 GML/LA, (**C**) 50/50 GML/LA, (**D**) 25/75 GML/LA, and (**E**) 0/100 GML/LA micelles. Circles denote intact vesicle adlayer properties prior to compound addition. Graphs are representative of at least three independent measurements per condition.

To further analyze the QCM-D data and corresponding structural transformation pathways, we plotted time-independent curves of the Δf vs. ΔD signals due to GML/LA mixture addition at 2× CMC (Figure 6). The complex, multi-step interaction profiles observed in all cases pointed to extensive vesicle disruption as opposed to strictly vesicle desorption. The addition of GML alone or 75/25 GML/LA caused large-scale membrane remodeling processes associated with budding-like behavior, while the nonlinear changes indicate a structural transformation in the vesicle adlayer properties due to membrane disruption [47,48]. More precisely, vesicle swelling occurred first, as indicated by increasing Δf and ΔD signals. Then, a point of critical instability in the vesicle adlayer properties was reached that resulted in vesicle rupture and partial loss of adsorbed lipid molecules from the sensor surface. Interestingly, the 50/50 GML/LA mixture caused a more complex pattern of large-scale structural transformations in the adlayer properties, as indicated by multiple slope changes in various directions due to competing membrane remodeling processes. In contrast to the preceding two cases, the initial interaction caused a decrease in the Δf signal before larger-scale changes in the Δf and ΔD signals occurred that corresponded to budding-like behavior. Eventually, a point of critical instability in the vesicle adlayer properties was also reached, leading to vesicle rupture. A similar pattern of interaction behavior occurred in the 25/75 GML/LA case, but the extent of membrane remodeling was smaller (i.e., Δf and ΔD shifts of less than −175 Hz and 50 × 10^−6^, respectively, in this case vs. Δf and ΔD shifts of greater than −200 Hz and 60 × 10^−6^, respectively, in the other cases described above). This finding supports a more direct pathway to vesicle disruption in the 25/75 GML/LA case. By contrast, the addition of LA alone caused vesicle disruption without large-scale membrane remodeling, which is consistent with the high strain induced in the outer leaflet, but the rupture process was incomplete in the LA case compared to the 25/75 GML/LA case.

As such, the high vesicle disruption activity of the 25/75 GML/LA mixture compared to other GML/LA mixtures and GML and LA alone is related to two factors working in parallel: (1) the relatively high fraction of LA causing enhanced instability in the outer leaflet of the vesicle bilayer due to anisotropic spontaneous curvature, and (2) competition between GML- and LA-induced membrane morphological changes further causing vesicle instability overall. Indeed, GML and LA are known to cause different types of membrane morphological changes since they mainly induce isotropic and anisotropic spontaneous curvature, respectively [19]. Whereas factor (2) alone appears to be the main contributor to synergistic membrane disruption observed in planar SLBs, the interplay of factors (1) and (2) is particularly important for vesicle disruption, especially considering the high-curvature membrane nanoarchitecture of the vesicle bilayer. 

From a broader perspective, we may also briefly comment on the vesicle rupture efficiency of the 25/75 GML/LA mixture compared to SDS, which is widely known as a membrane solubilizer [49,50]. While 1600 µM SDS micelles have been previously reported to exhibit ~50% rupture efficiency of ~70 nm diameter vesicles during the interaction step and ~60% rupture efficiency after a subsequent buffer washing step [22], the 25/75 GML/LA micelle treatment (at 260 μM concentration, an ~6-time lower concentration than in the SDS case) had ~67% and ~100% rupture efficiency values during the interaction step and after a subsequent buffer washing step, respectively. By contrast, LA itself had vesicle rupture efficiency values of ~34% and ~85% during the interaction step and after a subsequent buffer washing step, while GML had corresponding rupture efficiency values of ~37% and ~56%. Thus, by both vesicle rupture efficiency metrics, the 25/75 GML/LA mixture outperformed LA or GML alone, as well as SDS.

## 4. Conclusions

In this study, we have investigated the potential utility of employing antimicrobial lipid mixtures to disrupt small lipid vesicles within the size range of numerous enveloped virus types. While detergent-mediated virus inactivation is the main method of inhibiting enveloped viruses in the membrane disruption context [51,52], our study was motivated by the inability of the widely used SDS detergent to completely rupture adlayers composed of ~70 nm lipid vesicles in a past QCM-D study, whereas the complete rupture of ~120 nm lipid vesicles by SDS had been reported [22]. By comparing the membrane-disruptive effects of GML and LA vs. GML/LA mixtures, in the present study, we were able to ascertain that GML/LA mixtures can be designed to efficiently rupture small lipid vesicles of ~75 nm diameter to a greater extent than GML or LA alone. Of note, we identified that 25/75 GML/LA micelles exhibited the highest vesicle disruption performance, achieving a rupture efficiency value of 100% that indicated complete membrane solubilization. 

From an application perspective, this result was significant because it demonstrated that tuning the specific composition of GML/LA micelles can have a marked effect on modulating the degree of membrane disruption. From a biophysical perspective, this result was also striking because it further supports that membrane nanoarchitecture plays an important role in affecting the membrane-disruptive properties of antimicrobial lipid mixtures. Indeed, it was previously reported that a 50/50 GML/LA mixture had the greatest membrane-disruptive behavior towards a planar DOPC SLB [18], while our findings show that the 25/75 GML/LA mixture is superior for small, highly curved DOPC lipid vesicle disruption. We discussed this effect in terms of how nonionic GML and anionic LA differentially induce spontaneous curvature in lipid membranes, and such insights can be used to precisely engineer antimicrobial lipid mixtures with tailored membrane interaction profiles, especially when the experimental results are obtained using biomimetic lipid membrane systems that mimic critical features of the application target, such as intact vesicle adlayers for studying membrane curvature. In future work, it could be possible to further investigate how antimicrobial lipid mixtures interact with virus-mimicking model systems that possess more complex lipid compositions as well as with membrane-enveloped biological nanoparticles such as authentic virus particles and exosomes, and to establish biological correlates with functional properties such as virus inactivation.

## Data Availability

The raw data required to reproduce these findings are available from the corresponding authors on reasonable request.
